# High CIP2A levels correlate with an antiapoptotic phenotype that can be overcome by targeting BCL-X_L_ in chronic myeloid leukemia

**DOI:** 10.1038/leu.2016.42

**Published:** 2016-03-18

**Authors:** C M Lucas, M Milani, M Butterworth, N Carmell, L J Scott, R E Clark, G M Cohen, S Varadarajan

**Affiliations:** 1 Department of Molecular and Clinical Cancer Medicine, University of Liverpool, Liverpool, UK; 2 Department of Molecular and Clinical Pharmacology, University of Liverpool, Liverpool, UK

## Abstract

Cancerous inhibitor of protein phosphatase 2A (CIP2A) is a predictive biomarker of disease progression in many malignancies, including imatinib-treated chronic myeloid leukemia (CML). Although high CIP2A levels correlate with disease progression in CML, the underlying molecular mechanisms remain elusive. In a screen of diagnostic chronic phase samples from patients with high and low CIP2A protein levels, high CIP2A levels correlate with an antiapoptotic phenotype, characterized by downregulation of proapoptotic BCL-2 family members, including BIM, PUMA and HRK, and upregulation of the antiapoptotic protein BCL-X_L_. These results suggest that the poor prognosis of patients with high CIP2A levels is due to an antiapoptotic phenotype. Disrupting this antiapoptotic phenotype by inhibition of BCL-X_L_ via RNA interference or A-1331852, a novel, potent and BCL-X_L_-selective inhibitor, resulted in extensive apoptosis either alone or in combination with imatinib, dasatinib or nilotinib, both in cell lines and in primary CD34^+^ cells from patients with high levels of CIP2A. These results demonstrate that BCL-X_L_ is the major antiapoptotic survival protein and may be a novel therapeutic target in CML.

## Introduction

Chronic myeloid leukemia (CML) is a malignant disease of a primitive hematopoietic cell, characterized by a reciprocal translocation between chromosomes 9 and 22 and creates the fusion gene *BCR-ABL1*, which is a deregulated tyrosine kinase that drives the leukemia.^1^ CML treatment has been significantly improved by the tyrosine kinase inhibitor (TKI) imatinib, but some patients will eventually fail imatinib treatment and without a change in therapy, a significant proportion will progress towards blast crisis (BC), which is usually rapidly fatal.^2, 3^ The kinase activity of BCR-ABL is opposed by cellular phosphatases, such as protein phosphatase 2A (PP2A), which is impaired in several malignancies. PP2A plays an important role in regulating cell proliferation, differentiation and apoptosis. In CML, PP2A is inhibited by SET^4^ and cancerous inhibitor of PP2A (CIP2A).^5^ CIP2A inhibits PP2A activity and functions by preventing PP2A-driven dephosphorylation and stabilization of c-Myc.^6, 7, 8^ CIP2A is a strong prospective predictor of subsequent development of BC in imatinib-treated CML patients,^5^ although the underlying mechanisms remain unclear.

Apoptosis induction is tightly regulated by the BCL-2 family of proteins, which comprise several antiapoptotic members, such as BCL-2, BCL-X_L_, MCL-1, BCL-w and BFL-1, together with proapoptotic molecules, such as the multidomain effectors BAX and BAK, as well as the BH3-only proteins, including the activators BIM, BID and PUMA, and sensitizers NOXA, HRK, BIK, BMF and BAD.^9, 10^ The BH3-only members can either be promiscuous or selective with respect to binding their antiapoptotic counterparts. The activators bind all antiapoptotic BCL-2 family members, whereas the sensitizers NOXA and HRK are more selective in binding MCL-1 and BCL-X_L_, respectively.^11^ BCR-ABL modulates the expression levels and/or the phosphorylation status of several BCL-2 family members, thus exerting important regulatory effects on apoptosis.^12, 13, 14, 15^ Furthermore, recent reports suggest important roles for several antiapoptotic BCL-2 family members in CML disease progression.^16, 17, 18, 19, 20^ Elevated levels of these proteins in several cancers make them promising targets for drug therapy. Small-molecule inhibitors targeting specific members of the BCL-2 family, such as navitoclax/ABT-263 (BCL-2, BCL-X_L_ and BCL-w-specific) and venetoclax/ABT-199 (BCL-2-specific) are in clinical trials for several lymphoid malignancies.^21, 22^ Recently, selective inhibitors of BCL-X_L_ (A-1331852) and MCL-1 (A-1210477) have been synthesized.^23, 24^ These inhibitors target the antiapoptotic BCL-2 family members, displacing their sequestered proapoptotic counterparts, thereby resulting in apoptosis.

In this study, we demonstrate both a novel antiapoptotic role for CIP2A in CML pathogenesis and a key role for BCL-X_L_ in survival of CML cell lines and in primary CD34^+^ cells from patients. These results raise the possibility that inhibition of BCL-X_L_ may be a novel therapeutic option in CML, especially in patients refractory to TKI therapy.

## Materials and methods

### Reagents and antibodies

Imatinib, nilotinib and dasatinib were from Selleck Chemicals (Houston, TX, USA). ABT-737, ABT-199, A-1331852 and A-1210477 were kindly provided by AbbVie (North Chicago, IL, USA). Antibodies against BIM, PUMA, BMF, BIK, BAD, BCL-X_L_ and BCL-w were from Cell Signaling Technology (Danvers, MA, USA), GAPDH and MCL-1 from Santa Cruz Biotechnology (Santa Cruz, CA, USA), NOXA from Calbiochem (Darmstadt, Germany), BCL-2 from Dako (Ely, UK), BAX and BAK from Millipore (Watford, UK), HRK from Aviva Systems Biology (San Diego, CA, USA) and BID and BFL-1 were from Prof J Borst (The Netherlands Cancer Institute, Amsterdam, The Netherlands). All other reagents, unless mentioned otherwise, were from Sigma-Aldrich (St Louis, MO, USA).

### Patient cohort

The study was approved by the Liverpool Central Research Ethics Committee; all 31 patients gave informed consent and were aged 18 or over. All have been seen since original diagnosis of chronic phase CML at our center and have been followed for at least 12 months (median follow-up: 39 months). Patients' characteristics are presented in Supplementary Table S1.

### Sample collection, preparation and cell culture

At diagnosis, mononuclear cells from chronic phase CML patients were separated by density-dependent centrifugation (Lymphoprep Axis-Shield, Oslo, Norway), washed in RPMI 1640 (BioSera, Uckfield, UK) and resuspended in 10% dimethyl sulfoxide/10% fetal calf serum (BioSera)/RPMI at 4 °C and cryopreserved in liquid nitrogen. Wherever possible, samples were enriched for CD34^+^ cells using the CliniMACS kit (Miltenyi Biotec, Auburn, CA, USA). CD34^+^ cells were cultured using StemSpan SFEMII media (Stemcell Technologies, Cambridge, UK). K562 and KCL22 cells were cultured in RPMI 1640 supplemented with 10% fetal calf serum and 5 mm l-glutamine.

### BH3 profiling and flow cytometry

BH3 profiling was carried out using BH3 peptides from New England Peptide (Gardner, MA, USA) as previously described.^25^ Loss of mitochondrial membrane potential and apoptosis were quantified by flow cytometry as described.^26^ Patients with CIP2A levels ⩾7.3 mean fluorescence units by flow cytometry were defined as high CIP2A patients, as every patient that progressed to BC had CIP2A >7.3 mean fluorescence units.^27^ This cutoff value was derived using receiver operating characteristics (ROC) curve analysis for the prediction of BC based on the diagnostic CIP2A protein level; minimization of the Euclidian distance between the receiver operating characteristics curve and the corner (0, 1) was the criterion used. The optimal cutoff value produced an AUC_ROC_=0.902 (95% CI: 0.832, 0.973).

### siRNA knockdowns, immunoprecipitation and western blotting

Cells were reverse-transfected with 10 nm of BAK (s1880 and s1881), BAX (s1888 and s1889), BIM (s195011), PUMA (pool of siRNAs), BMF (pool of siRNAs), BIK (s1989 and s1990), HRK (s194952), BCL-X_L_ (s1920), MCL-1 (s8583), BCL-w (s1924), BFL-1 (pool of siRNAs) from Life Technologies (Paisley, UK), BID (SI02654568), NOXA (SI00129430), BAD (SI00299348), BCL-2 (S100299411) from Qiagen (Manchester, UK) using Interferin (Polyplus Transfection, NY, USA), according to the manufacturer's protocol and processed 48 h after transfection. Immunoprecipitation and western blotting were carried out according to the standard protocols.^26^

### mRNA expression

Quantitative reverse transcription–PCR was performed using cDNA from total leukocytes. Pre-designed TaqMan real-time PCR assays were used for *BCL2L11* (Hs00708019_s1), *BCL2L1* (Hs00236329_m1), *BID* (Hs00609632_m1), *BBC3* (Hs00248075_m1), *HRK* (Hs02621354_s1) and *BAD* (Hs00188930_m1) and *GAPDH* (Hs99999905_m1) (Life Technologies). PCR was performed using a Stratagene MX3005P PCR machine (Agilent Technologies, Folsom, CA, USA). In evaluating the mRNA expression data, the comparative Ct method was used, with the 2^−ΔΔCt^ formula to achieve results for relative quantification. A pool of cDNA from four normal individuals was used as a calibrator and all samples were normalized to *GAPDH*.

### Statistical analysis

Statistical analysis was conducted using one-way analysis of variance applying the Welch correction and Dunnet's two-sided multiple comparison test to compare the different treatments to the appropriate control peptide/siRNA (*P⩽0.05, **P⩽0.01, ***P⩽0.001). For continuous variables, the Mann–Whitney *U-*test was used for comparisons between independent samples. For categorical variables, Fisher's exact test was used. Progression-free survival functions were estimated by the Kaplan–Meier estimator and the log-rank test was used for comparisons between groups. Statistical analysis was performed using GraphPad Prism (GraphPad Prism Software, Inc., La Jolla, CA, USA).

## Results

### TKIs prime CML cell lines to undergo apoptosis

**Figure 1:**
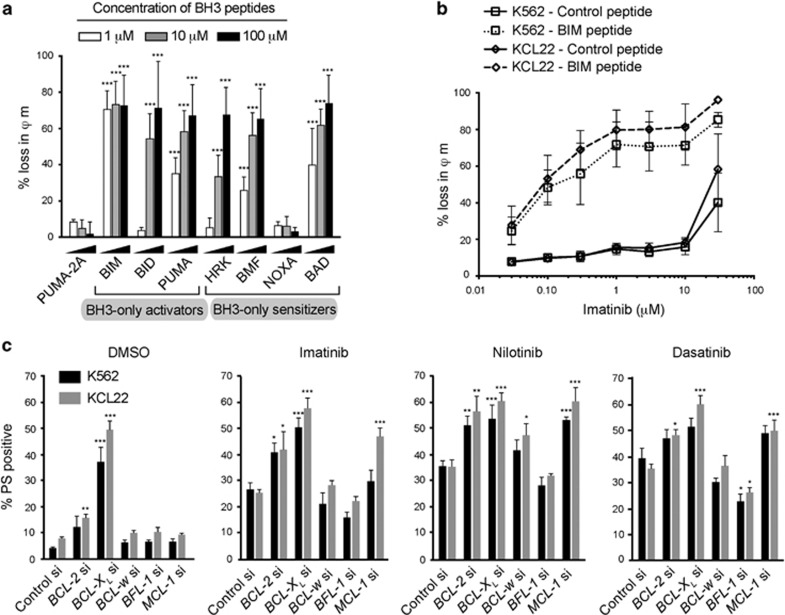
BH3 profiling and RNA interference implicate roles for BCL-2, BCL-X_L_ and MCL-1 in TKI-induced apoptosis. (**a**) BH3 profiling in K562 cells was carried out using the specified concentrations of different BH3 peptides for 2 h. PUMA-2A was used as the control peptide. (**b**) Dynamic BH3 profiling in K562 and KCL22 cells, exposed to increasing concentrations of imatinib for 16 h, was carried out using either control peptide (bold continuous lines) or BIM peptide (dotted lines) at 1 μm for 2 h. (**c**) K562 and KCL22 cells, reverse-transfected with the indicated siRNAs, were exposed for 48 h to dimethyl sulfoxide, imatinib (1 μm), nilotinib (50 nm) or dasatinib (3 nm) and apoptosis assessed by phosphatidylserine (PS) externalization. Statistical analysis was conducted using one-way analysis of variance applying the Welch correction and Dunnet's two-sided multiple comparison test to compare the different treatments to the appropriate control peptide/ siRNA (*P⩽0.05, **P⩽0.01, ***P⩽0.001). Error bars represent standard error of mean (s.e.m.) from three independent experiments.

Since high levels of CIP2A contributed to imatinib resistance in CML, we wished to understand the role of BCL-2 family members in this resistance mechanism. Using BH3 profiling, a peptide-based technique to determine BCL-2 family dependencies,^25^ we observed extensive loss of mitochondrial membrane potential (*ϕ*_m_) in two CML cell lines, K562 and KCL22, following exposure to increasing concentrations of different BH3 peptides (Figure 1a and Supplementary Figure S1). Although all BH3-only activators exhibited extensive mitochondrial depolarization, BH3-only sensitizers demonstrated greater selectivity as demonstrated by a concentration-dependent loss in *ϕ*_m_ following BMF, BAD and HRK, but not NOXA (Figure 1a and Supplementary Figure S1). These results suggested that the survival of these cells depended more on BCL-2, BCL-X_L_ and BCL-w, rather than on MCL-1 and BFL-1, as NOXA was the only sensitizer among the list to specifically target both MCL-1 and BFL-1 (Figure 1a and Supplementary Figure S1).^9, 10, 11^ In dynamic BH3 profiling studies,^28^ increasing concentrations of TKIs resulted in a significant loss of *ϕ*_m_, only when the cells were subsequently exposed to the BIM peptide, suggesting that TKIs primed these cells to apoptosis and a combination therapy with another apoptotic stimuli could facilitate rapid apoptosis in these cells (Figure 1b and Supplementary Figure S2). Since our data implicated specific members of the BCL-2 family in antagonizing apoptosis, we performed RNA interference to silence the expression of different BCL-2 family members to study their effects on TKI-mediated apoptosis (Figure 1c). The concentrations of TKIs used in these studies were determined from their concentration–response curves (Supplementary Figure S3). Downregulation of BCL-X_L_ and to some extent BCL-2 resulted in apoptosis, suggesting that BCL-X_L_ is a critical survival factor in both CML cell lines (Figure 1c). Furthermore, downregulation of BCL-X_L_, and to a lesser extent, BCL-2 and MCL-1, significantly potentiated TKI-mediated apoptosis in both K562 and KCL22 (Figure 1c), thus confirming an important role for antiapoptotic BCL-2 family members in TKI-mediated apoptosis.

### TKIs induce apoptosis in a BH3-dependent manner

**Figure 2:**
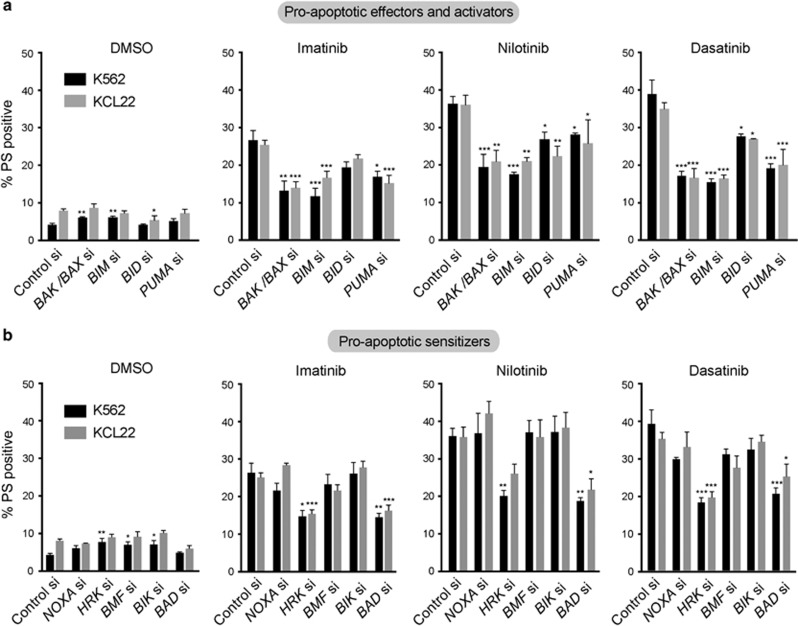
TKIs induce apoptosis in a BH3-dependent manner. (**a**, **b**) K562 and KCL22 cells, reverse-transfected with the indicated siRNAs, were exposed for 48 h to dimethyl sulfoxide, imatinib (1 μm), nilotinib (50 nm) and dasatinib (3 nm) and apoptosis assessed by phosphatidylserine (PS) externalization. Statistical analysis was conducted using one-way analysis of variance applying the Welch correction and Dunnet's two-sided multiple comparison test to compare the different siRNA transfections to their appropriate control siRNA in each treatment (*P⩽0.05, **P⩽0.01, ***P⩽0.001). Error bars represent s.e.m. from three independent experiments.

Exposure to TKIs caused a time-dependent decrease in the expression levels of most anti- and proapoptotic BCL-2 family members, with the notable exception of BAD, which was significantly upregulated (Supplementary Figure S4). To understand the relative contribution of different proapoptotic BCL-2 members in TKI-mediated apoptosis, we silenced the expression of BAX, BAK as well as BH3-only activators and sensitizers in K562 and KCL22 (Figure 2). Although all the proapoptotic effector and activator proteins were critical for TKI-mediated apoptosis, a selective dependence on HRK and BAD, but not NOXA, BMF or BIK was observed in TKI-mediated apoptosis (Figure 2 and Supplementary Figure S4), thus implicating a regulatory role for several BH3-only members in TKI-induced apoptosis.

### Downregulation of proapoptotic BCL-2 family proteins is associated with disease progression in imatinib-treated patients

**Figure 3:**
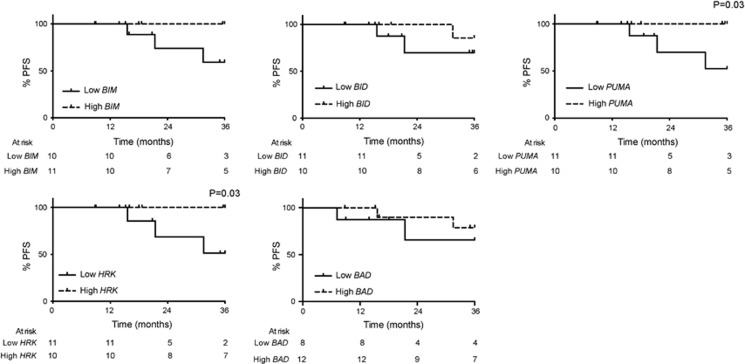
Expression levels of the proapoptotic BH3-only proteins correlate with progression-free survival in CML patients. Progression-free survival for patients treated with imatinib at initial diagnosis. PCR was performed using total leukocytes collected at initial diagnosis. Patients were stratified into high and low expression groups according to the median mRNA expression for *BIM, BID, PUMA, HRK* and *BAD* and the number of cases assessed presented below each graph. The log-rank test was used to determine the significance between high and low expressers.

To investigate a possible relationship between these BH3-only proteins and clinical outcome, we compared the mRNA expression levels of these proteins with progression-free survival of chronic phase CML patients, treated with imatinib at diagnosis (Figure 3). The median expression level for each gene was calculated and patients were stratified as high or low relative to the median. Low *BIM* expression was associated with an inferior progression-free survival, whereas *BID* or *BAD* expression did not correlate with clinical outcome (Figure 3). Low *PUMA* and *HRK* expression were significantly associated with disease progression to BC (*P*=0.03; Figure 3). In this study, four patients progressed to BC and this disease progression was not associated with the presence of BCR-ABL kinase domain mutations. Low expression of *BIM, PUMA* and *HRK* was also associated with poor overall survival but this did not reach significance (data not shown). Moreover, 50% of patients with low *PUMA* or *HRK* expression at diagnosis had progressed by 36 months (Figure 3). In addition, low diagnostic levels of *BIM* and *HRK* were associated with a slower rate of deep molecular response (MR5) during the first three years of treatment (data not shown).

### CIP2A levels correlate with the balance between pro- and antiapoptotic BCL-2 family proteins

**Figure 4:**
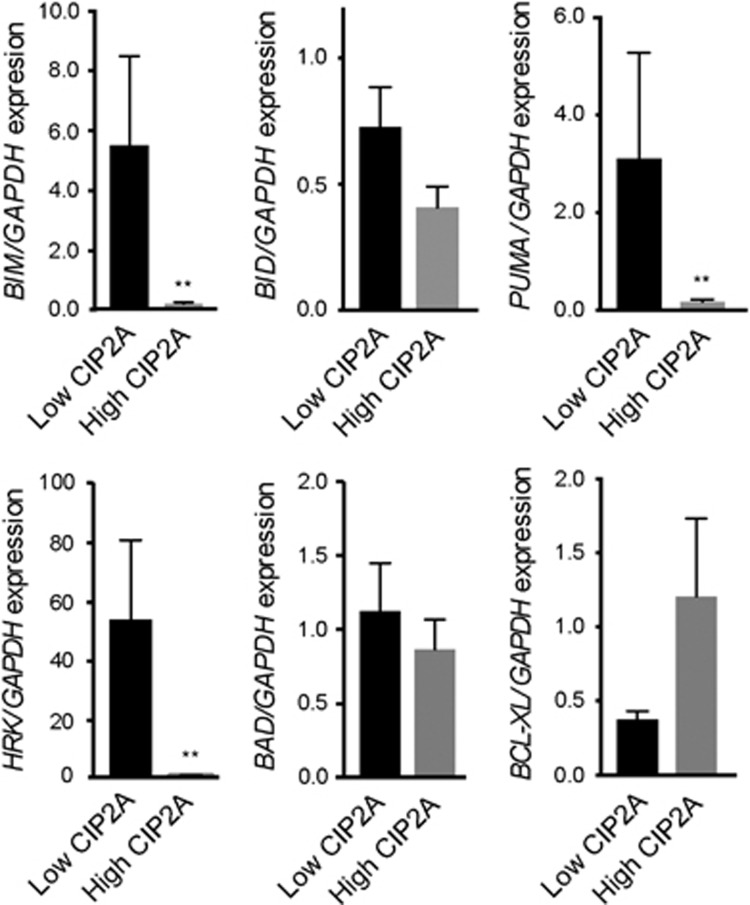
High CIP2A expression levels correlate with an antiapoptotic phenotype. mRNA expression for *BIM*, *BID, PUMA*, *HRK*, *BAD* and *BCL-X*_L_ in 31 newly diagnosed chronic phase CML patients stratified by their diagnostic CIP2A status. A pool of four normal healthy volunteers was used as a calibrator pool. Statistical analysis was conducted using a Mann–Whitney *U-*test comparing high and low CIP2A patients (**P⩽0.01). Error bars represent s.e.m.

Since CML disease progression correlates with high CIP2A levels,^5, 29^ as well as changes in expression levels of different BCL-2 family members (Figure 3), we speculated whether CIP2A levels could correlate with the expression levels of different BCL-2 family members. To investigate this possibility, we assessed mRNA expression for *BIM*, *BID, PUMA*, *HRK*, *BAD* and *BCL-X_L_* in newly diagnosed chronic phase CML patients. Expression levels of *BIM*, *PUMA* and *HRK* were significantly lower in high compared with low CIP2A patients (Figure 4). A similar trend was observed for *BID* and *BAD* expression but this did not reach statistical significance (Figure 4). In contrast, patients with high CIP2A levels expressed high levels of *BCL-X*_L_, although this did not reach statistical significance (Figure 4). Taken together these results suggest that CIP2A may exhibit its oncogenic activity by altering the balance of pro- and antiapoptotic proteins resulting in an antiapoptotic phenotype.

### BCL-X_L_ is a critical survival factor and antagonizes TKI-induced apoptosis in CML cell lines

**Figure 5:**
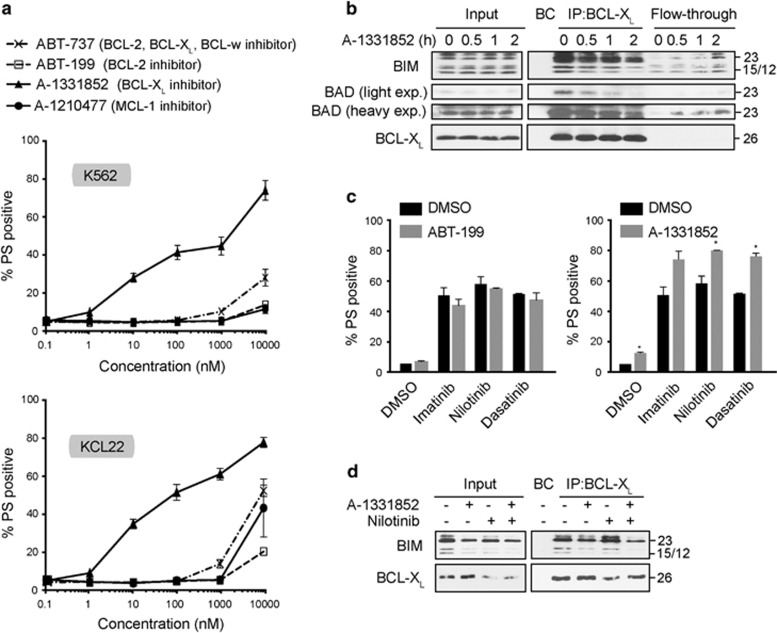
BCL-X_L_ is a critical survival factor and regulates TKI-induced apoptosis in CML cell lines. (**a**) K562 and KCL22 cells were exposed for 24 h to the specified inhibitors and apoptosis assessed by phosphatidylserine (PS) externalization. (**b**) Immunoprecipitation of BCL-X_L_ was carried out in K562 cells, exposed to A-1331852 (100 nm) for 0–2 h, and the eluted complexes were immunoblotted for the indicated proteins. The input cell lysates and the immunodepleted supernatant (labeled as Flow-through) were immunoblotted to check the efficiency of the immunoprecipitation. BC represents beads control. (**c**) K562 cells, exposed for 1 h to 1 nm of ABT-199 or A-1331852, were further exposed in the presence of the pretreated inhibitors to imatinib (1 μm), nilotinib (50 nm) or dasatinib (3 nm) for 24 h and apoptosis assessed. (**d**) Same as (**b**) but the immunoprecipitation was carried out with antibodies against BCL-X_L_ in K562 cells exposed to A-1331852 (1 nm) with or without nilotinib (50 nm) for 24 h. Statistical analysis was conducted using one-way analysis of variance applying the Welch correction and Dunnet's two-sided multiple comparison test to compare the TKI treatments with the combination treatments of A-1331852, represented by the black and gray histograms (*P⩽0.05). Error bars represent s.e.m. from three independent experiments.

Since our initial data identified BCL-X_L_ as a critical survival factor in CML cell lines, we used a toolkit of selective BCL-2 family inhibitors, comprising ABT-737 (BCL-2, BCL-X_L_ and BCL-w-specific inhibitor), ABT-199 (BCL-2-selective), A-1331852 (BCL-X_L_-specific) and A-1210477 (MCL-1-selective) to further evaluate the role of BCL-X_L_ in CML cell survival. In both cell lines, A-1331852 was extremely potent, inducing apoptosis at low nanomolar concentrations, whereas the other inhibitors failed to induce apoptosis even at 100-fold higher concentrations (Figure 5a). A-1331852 was efficient in displacing both BIM and BAD from BCL-X_L_ and releasing them into the cytosol (Figure 5b). Furthermore, A-1331852, but not ABT-199, was efficacious in potentiating TKI-mediated apoptosis for 2G TKIs (Figure 5c). A combination of nilotinib and A-1331852 was more potent than either A-1331852 or nilotinib alone in displacing BIM from BCL-X_L_ (Figure 5d), further suggesting that A-1331852 can be effective in inducing apoptosis in CML cell lines, either as a single agent or in combination with TKIs.

### A-1331852 exhibits remarkable potency both as a single agent and in combination with TKIs in killing primary CD34^+^ CML cells

**Figure 6:**
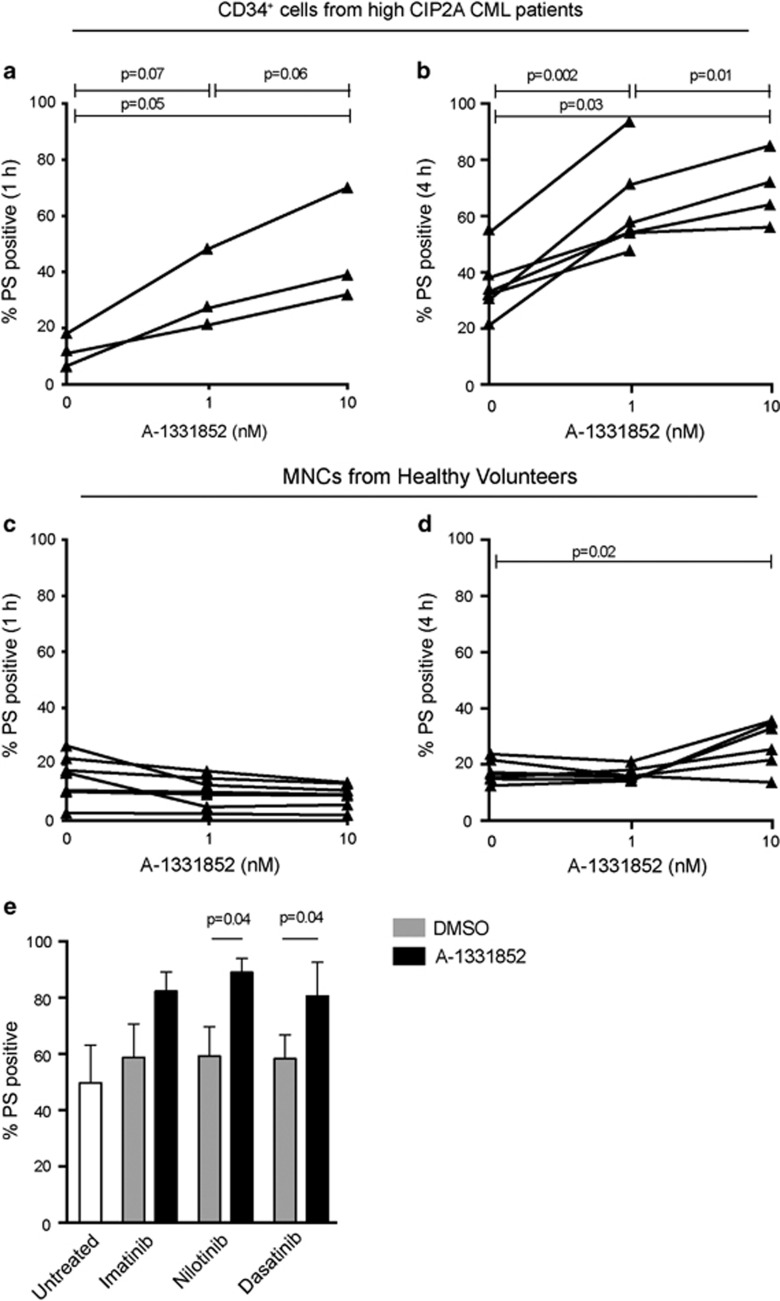
Inhibition of BCL-X_L_ promotes rapid apoptosis in primary CML cells. (**a**, **b**) Diagnostic chronic phase CD34^+^ cells from high CIP2A patients were exposed to A-1331852 for 1 h (*n=*3) and 4 h (*n=*5) and apoptosis assessed. (**c**, **d**) Mononuclear cells (MNCs) from healthy volunteers were exposed to A-1331852 for 1 h (*n=*8) and 4 h (*n=*6) and apoptosis assessed. (**e**) Diagnostic chronic phase CD34^+^ cells from high CIP2A patients were exposed to imatinib (5 μm), dasatinib (150 nm) and nilotinib (5 μm) for 24 h followed by the addition of A-1331852 (10 nm) to the cells for a further 1 h (*n=*5). Statistical analysis was conducted using a Mann–Whitney *U-*test and *P*-values specified, where significant. Error bars represent s.e.m.

**Figure 7:**
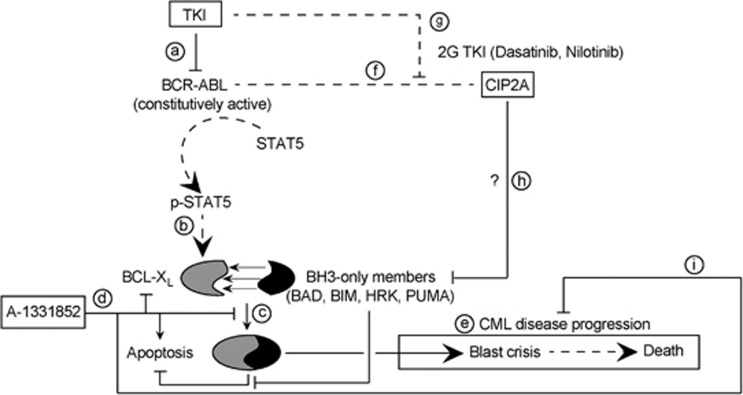
Selective inhibition of BCL-X_L_ overcomes CIP2A-mediated regulation of BCL-2 family members and disease progression in CML. The links that we have established/confirmed in this study are presented as bold lines whereas the dashed lines represent findings from literature. (a) The constitutively active kinase activity of BCR-ABL, antagonized by TKIs, results in phosphorylation of STAT5. (b) p-STAT5 induces the transcription of BCL-X_L_. (c) BCL-X_L_ sequesters and inhibits BH3-only proteins. (d) This antiapoptotic activity is abolished by A-1331852. (e) Failure to achieve effective apoptosis results in disease progression. (f) High levels of CIP2A correlate with imatinib resistance in CML patients. (g) This can be overcome by second generation (2G) TKIs. (h) CIP2A expression levels correlate with an antiapoptotic phenotype characterized by changes in the balance between the pro- and antiapoptotic BCL-2 family members, thus conferring resistance to TKI therapy in CML. The precise mechanisms by which high CIP2A correlates with the antiapoptotic phenotype is unknown and hence marked with a ‘?' (i) Selective inhibition of BCL-X_L_ induces rapid apoptosis in CML cells, thus providing a novel and promising therapeutic option.

We next investigated the ability of A-1331852 to induce apoptosis in primary CD34^+^ progenitor cells from high CIP2A patients. In agreement with our data in CML cell lines, A-1331852 displayed remarkable potency in inducing apoptosis in these cells at low nanomolar concentrations as early as 1 h post-treatment (Figure 6a). Prolonged exposure (4 h) resulted in improved potency as A-1331852 induced extensive apoptosis (*P*=0.002) at concentrations as low as 1 nm in these cells (Figure 6b). Similar results were observed in CD34^+^ progenitor cells from low CIP2A patients (Supplementary Figure S5). In contrast, mononuclear cells isolated from healthy volunteers generally remained insensitive to the treatment (Figures 6c and d). This is particularly significant as clinically achievable concentrations of imatinib (5 μm), nilotinib (5 μm) or dasatinib (150 nm) did not induce significant apoptosis in primary CD34^+^ cells after 4 h exposure (data not shown). Even after 24 h exposure, none of these TKIs induced much if any apoptosis above the high spontaneous apoptosis observed in the progenitor cells (Figure 6e). However a subsequent and short exposure to A-1331852 (1 h) following the initial 24 h exposure to TKIs was sufficient to induce enhanced apoptosis in these CD34^+^ cells (*P*⩽0.01, Figure 6e). These data support the possibility of targeting BCL-X_L_, as a novel and effective therapeutic strategy in CML (Figure 7).

## Discussion

High expression of CIP2A contributes to imatinib resistance in CML and is a strong prospective predictor of subsequent development of BC in imatinib-treated patients.^5^ However the mechanism(s) by which CIP2A increases the risk of disease progression is poorly understood. In this study, we have identified several proapoptotic BCL-2 family members to be critical in TKI-mediated apoptosis (Figures 1 and 2). These findings also extended to CML patients, as decreased expression of specific proapoptotic BH3-only members *PUMA*, *HRK* and possibly *BIM* correlated with disease progression in CML patients (Figure 3). To our knowledge, this is the first study to link several proapoptotic BCL-2 family members to progression-free survival in imatinib-treated CML patients. We show that high CIP2A expression levels correspond to low expression of specific BH3-only proteins, *BIM*, *PUMA* and *HRK*, and an increase in the expression of *BCL-X_L_* (Figure 4), highly characteristic of an antiapoptotic phenotype.

Recently, we have shown that administration of 2G TKIs, such as nilotinib and dasatinib, can overcome high CIP2A and prevent disease progression.^5, 27^ However, this is not without worrying side effects, as dasatinib has a 25% risk of pleural effusion within ~3 years and nilotinib therapy is associated with hyperglycemia in some patients and a dose-related (8–10%) risk of myocardial infarction, cerebrovascular event or peripheral arterial occlusive event by 6 years.^30, 31^ This necessitates research into possible alternate therapeutic strategies. In this study, using a BCL-X_L_-specific inhibitor, A-1331852,^23^ we demonstrate for the first time, an effective therapeutic option for CML patients with high CIP2A expression levels. A-1331852 displayed remarkable potency, both as a single agent and in combination with TKIs, to induce apoptosis in cell lines and in progenitor CD34^+^ primary cells (Figures 5 and 6) demonstrating the critical importance of BCL-X_L_ in the survival of CML cells. Although BCL-X_L_ has been associated with disease progression,^12, 19, 32, 33, 34^ this is the first study that demonstrates a novel antiapoptotic role for CIP2A in CML pathogenesis and how this can be overcome by selectively targeting BCL-X_L_. This therapeutic option appears particularly promising because the CD34^+^ progenitor cells were highly sensitive to nanomolar concentrations of A-1331852 but insensitive to even prolonged exposure of the TKIs (Figure 6). This observation is in agreement with previous studies demonstrating a BCL-X_L_ dependence of stem cell survival for human embryonic stem cells as well as non-small-cell lung cancer cells.^35, 36^ Thus, targeting BCL-X_L_ potentially offers great therapeutic benefits in CML, especially due to the insensitivity of quiescent CD34^+^ progenitor CML cells to imatinib, which is a major factor in the recurrence of the disease on discontinuation of therapy,^17, 37^ although it will be necessary to overcome potential toxicities, such as thrombocytopenia, associated with BCL-X_L_ inhibition.^22, 38^

In summary, we clearly demonstrate that high CIP2A corresponds to an antiapoptotic phenotype, which may contribute to the poor prognosis of CML patients. We have also shown that this antiapoptotic phenotype can be overcome in CML by targeting BCL-X_L_, thus identifying an effective therapeutic option for CML patients with high expression levels of CIP2A (Figure 7). As high CIP2A levels are also implicated in disease progression in acute myeloid leukemia, breast, bladder, cervical, colon, hepatocellular and lung cancer,^39, 40, 41, 42, 43, 44, 45, 46^ it will be of interest to ascertain if these tumors also exhibit an antiapoptotic phenotype. Targeting this antiapoptotic phenotype with selective BCL-2 family antagonists may offer novel therapeutic approaches to these malignancies.
