# Supplementary figures and images for: High CIP2A levels correlate with an antiapoptotic phenotype that can be overcome by targeting BCL-XL in chronic myeloid leukemia
Source: Leukemia. 2016 Mar 18;30(6):1273–81. doi: 10.1038/leu.2016.42 (PMC4895185; doi:10.1038/leu.2016.42)

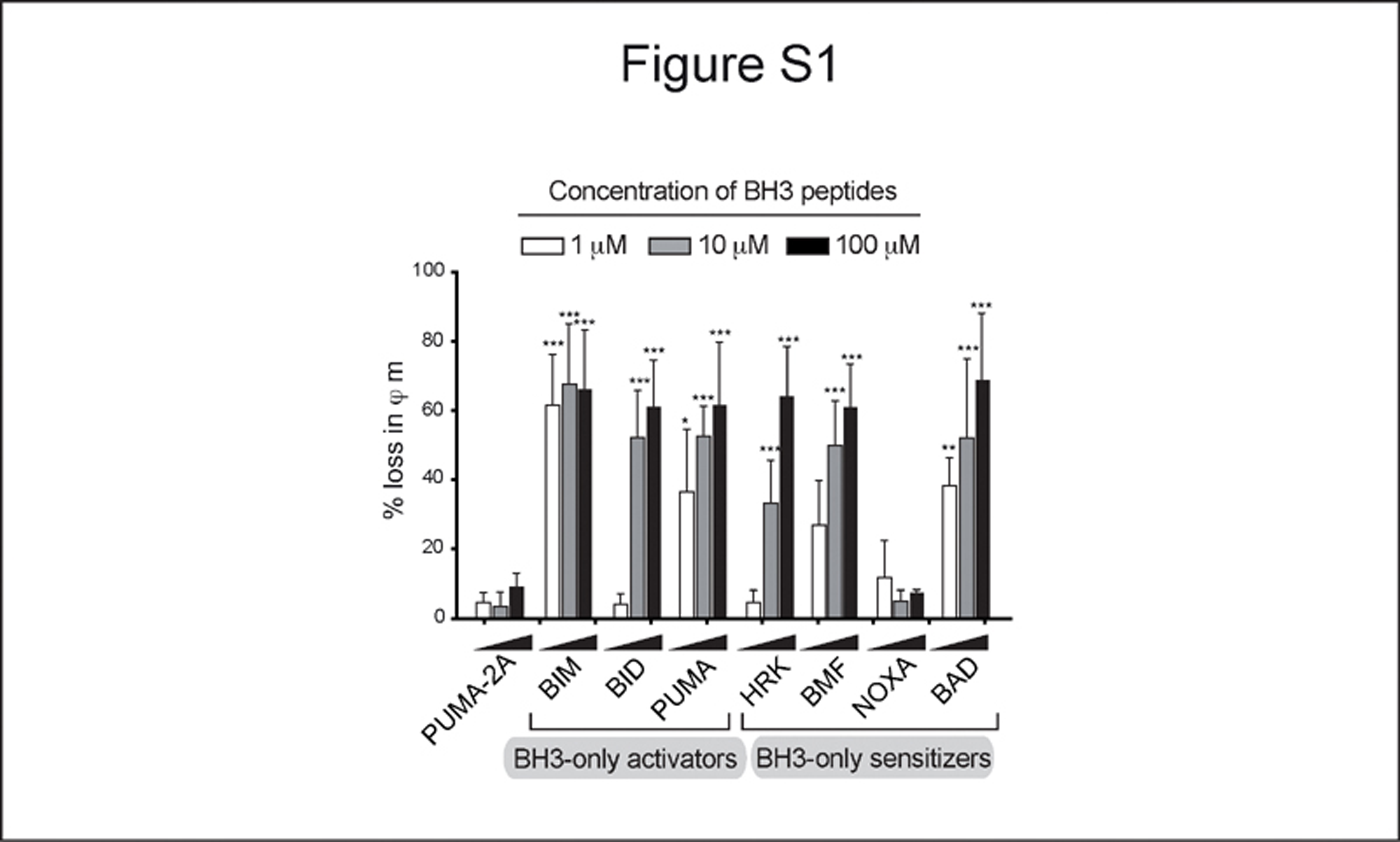

Supplement: Supplementary Figure S1 [file leu201642x1.tif]

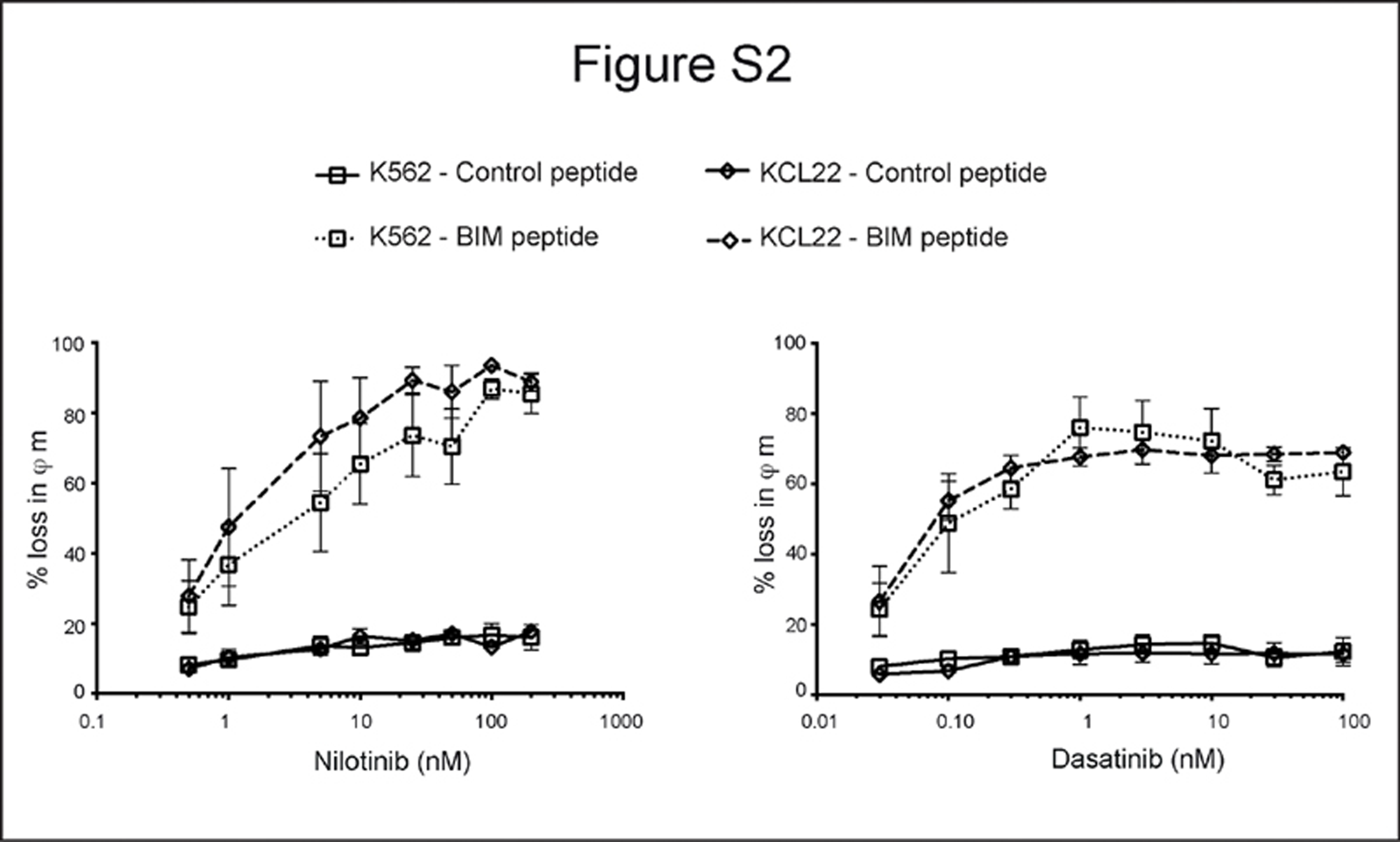

Supplement: Supplementary Figure S2 [file leu201642x2.tif]

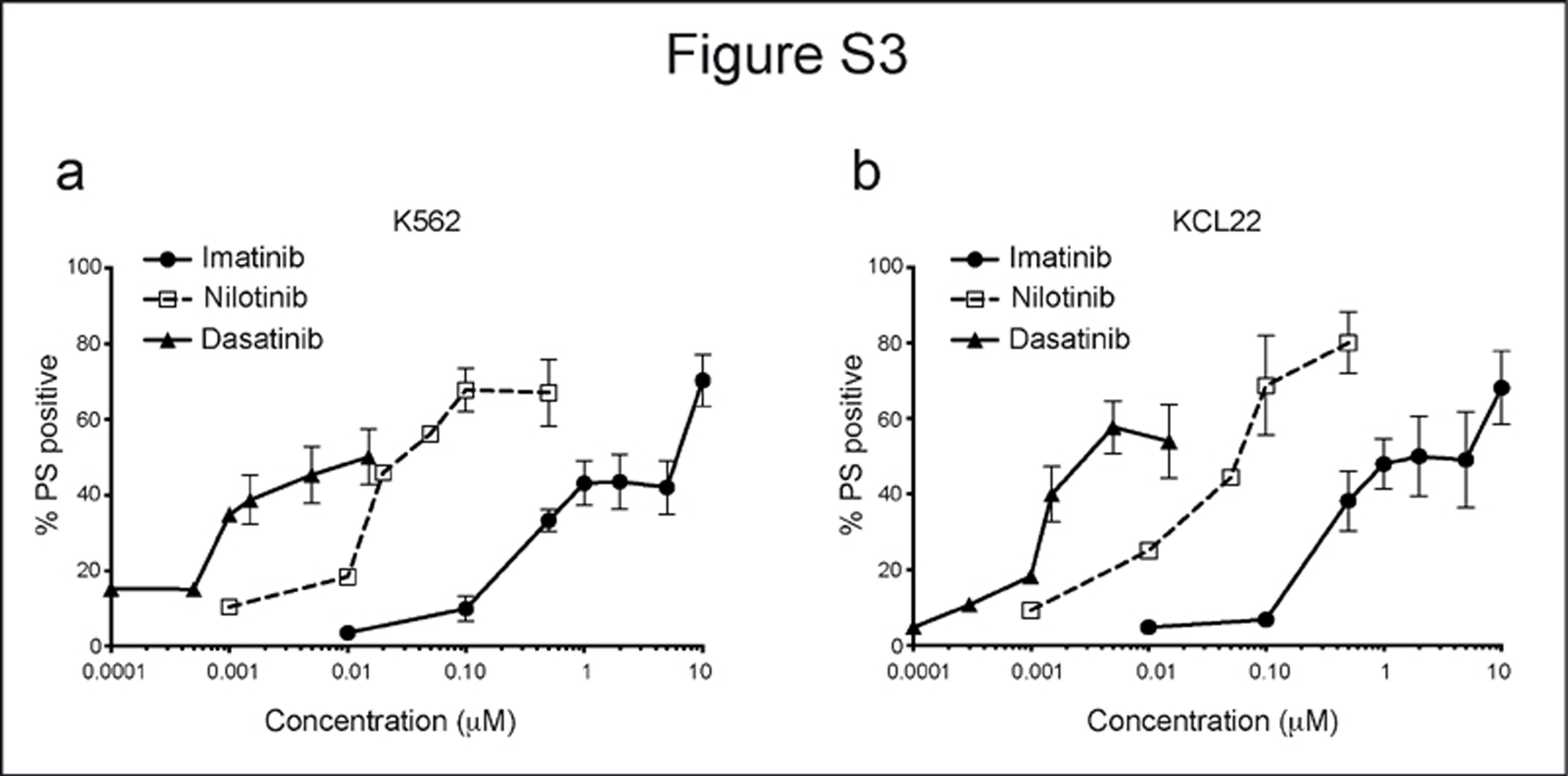

Supplement: Supplementary Figure S3 [file leu201642x3.tif]

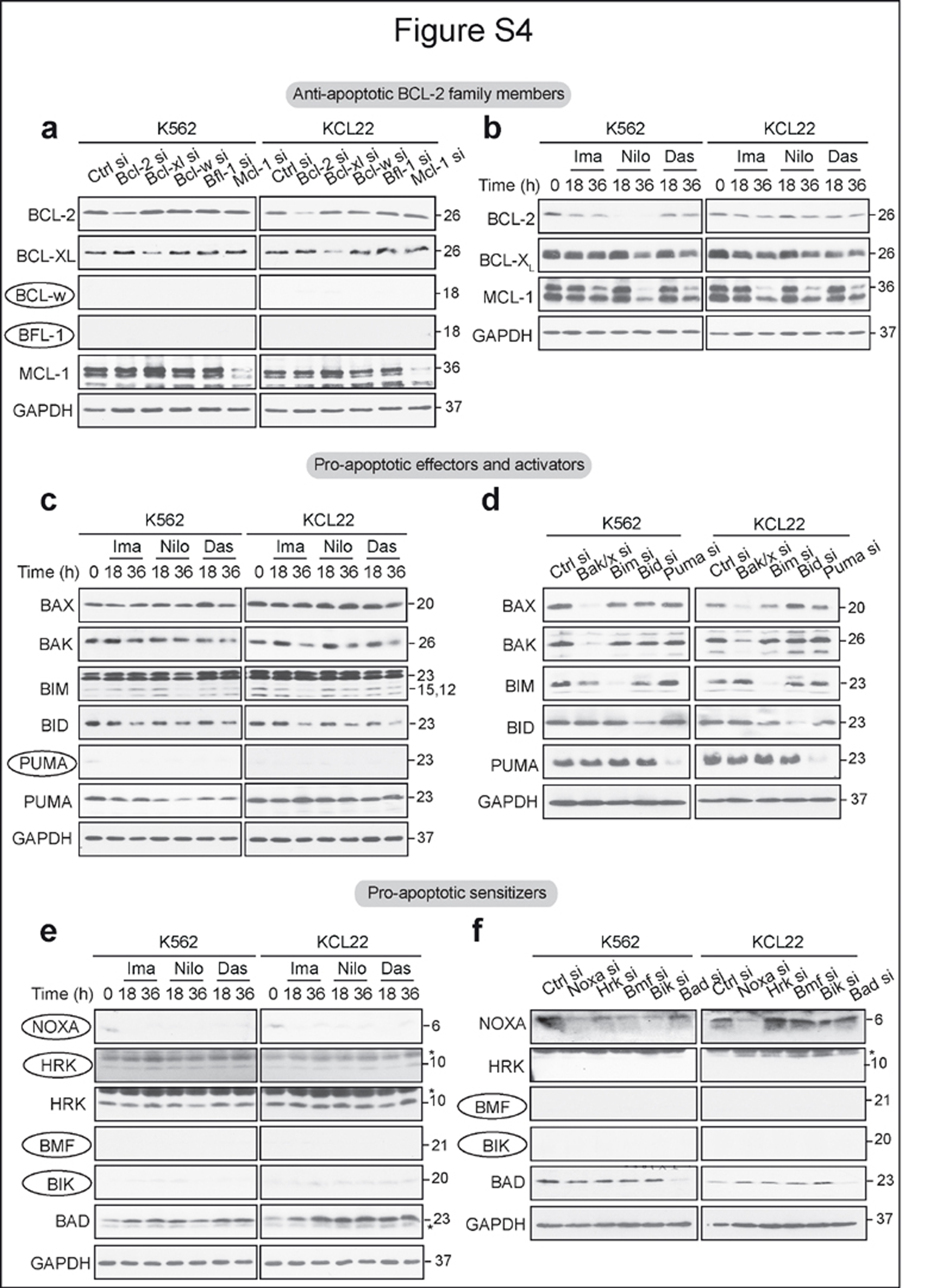

Supplement: Supplementary Figure S4 [file leu201642x4.tif]

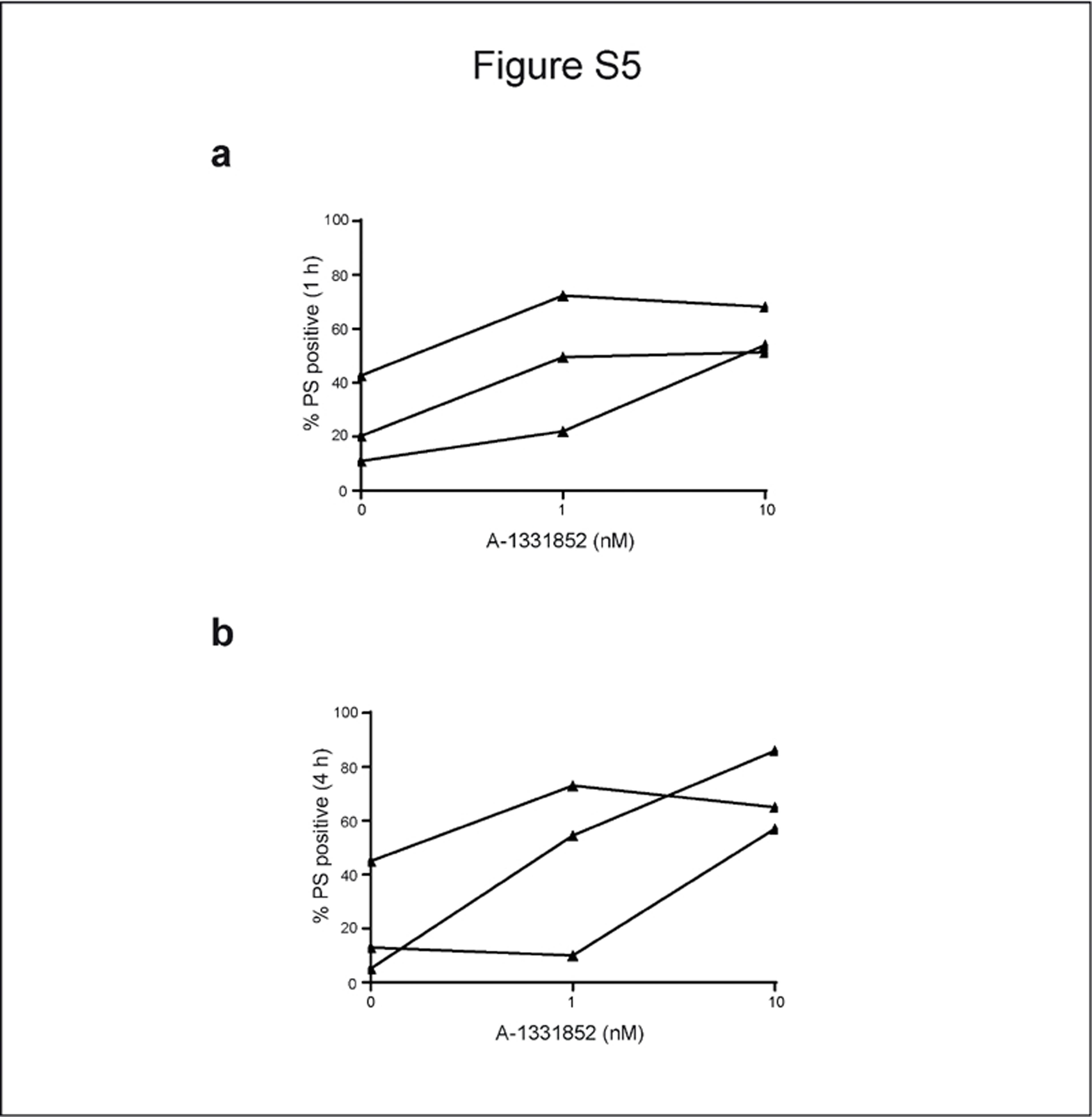

Supplement: Supplementary Figure S5 [file leu201642x5.tif]
