# Supplementary material for: High CIP2A levels correlate with an antiapoptotic phenotype that can be overcome by targeting BCL-XL in chronic myeloid leukemia
Source: Leukemia. 2016 Mar 18;30(6):1273–81. doi: 10.1038/leu.2016.42 (PMC4895185; doi:10.1038/leu.2016.42)
Supplement: Supplementary Table 1 [file leu201642x6.doc]

Supplementary Table 1

| **Patient ID** | **First line treatment** | **CIP2A status at diagnosis** | **Sex** | **Age at diagnosis** |
| --- | --- | --- | --- | --- |
| 1 | imatinib | Low | m | 73.6 |
| 10 | imatinib | Low | f | 54.2 |
| 30 | imatinib | Low | m | 48.6 |
| 31 | imatinib | Low | m | 48.4 |
| 85 | imatinib | Low | f | 18.9 |
| 107 | imatinib | Low | m | 40.0 |
| 115 | imatinib | high | m | 24.4 |
| 119 | imatinib | Low | m | 72.4 |
| 123 | imatinib | high | m | 64.5 |
| 124 | imatinib | Low | m | 33.7 |
| 206 | imatinib | Low | f | 43.4 |
| 218 | imatinib | Low | f | 45.8 |
| 220 | imatinib | high | m | 60.1 |
| 296 | imatinib | Low | m | 24.5 |
| 328 | imatinib | Low | f | 67.2 |
| 385 | imatinib | high | m | 32.6 |
| 395 | imatinib | high | f | 28.3 |
| 441 | imatinib | Low | m | 62.3 |
| 466 | imatinib | Low | f | 40.3 |
| 467 | imatinib | Low | m | 58.0 |
| 528 | imatinib | Low | f | 18.8 |
| 208 | 2G TKI | Low | m | 45.1 |
| 243 | 2G TKI | Low | m | 62.2 |
| 315 | 2G TKI | high | f | 47.9 |
| 437 | 2G TKI | high | f | 67.1 |
| 443 | 2G TKI | high | f | 73.2 |
| 464 | 2G TKI | high | f | 27.2 |
| 468 | 2G TKI | high | f | 53.4 |
| 481 | 2G TKI | high | m | 75.3 |
| 509 | 2G TKI | Low | f | 58.2 |
| 517 | 2G TKI | Low | m | 64.5 |
