# Supplementary material for: High CIP2A levels correlate with an antiapoptotic phenotype that can be overcome by targeting BCL-XL in chronic myeloid leukemia
Source: Leukemia. 2016 Mar 18;30(6):1273–81. doi: 10.1038/leu.2016.42 (PMC4895185; doi:10.1038/leu.2016.42)
Supplement: Supplementary Legends [file leu201642x7.doc]

**Legends to supplementary tables and figures**

**Supplementary Table 1.** Patient Characteristics.

**Figure S1.** BH3 profiling implicates a role for different anti-apoptotic Bcl-2 family members in Tyrosine Kinase inhibitor (TKI)-induced apoptosis. BH3 profiling in KCL22 cells was carried out using 1, 10 or 100μM of different BH3 peptides for 2h. PUMA-2A was used as a negative control for BH3 peptides. The cells were then stained with tetramethylrhodamine ethyl ester (TMRE) and the extent of loss in mitochondrial membrane potential (ϕm) was assessed. The % loss of ϕm was calculated with reference to DMSO (0% depolarized) and FCCP (100% depolarized) treated cells. Error bars represent the standard error of mean (SEM) from at least three independent experiments.

**Figure S2.** TKIs prime CML cell lines to apoptosis. K562 and KCL22 cells, exposed to increasing concentrations of nilotinib and dasatinib for 16h, were permeabilized with 0.002% digitonin, followed by exposure to either control peptide (bold continuous lines) or BIM peptide (dotted lines) at 1μM for 2h. The cells were then stained with TMRE and the extent of loss in ϕm assessed. Error bars represent SEM from at least three independent experiments.

**Figure S3.** TKIs induce apoptosis in a concentration-dependent manner in both CML cell lines. **(a** and **b)** K562 and KCL22 cells were exposed for 48h to various concentrations of tyrosine kinase inhibitors, imatinib (bold lines with filled circle), nilotinib (dotted lines with hollow square) and dasatinib (bold lines with filled triangle) and the extent of apoptosis assessed by PS externalization. In the graph, the extent of apoptosis in untreated control cells matched the % apoptosis of the lowest concentration tested for all inhibitors. Error bars represent SEM from at least three independent experiments.

**Figure S4.** Exposure to TKIs alters expression levels of several BCL-2 family members in a time-dependent manner. (**a**) Whole cell lysates of K562 and KCL22 cells, reverse-transfected with siRNAs against the anti-apoptotic BCL-2 family members (BCL-2, BCL-X_L_, BCL-w, BFL-1 and MCL-1) for 48h, were immunoblotted with the indicated antibodies to assess transfection efficiency. The labels of anti-apoptotic members, BCL-w and BFL-1 are encircled to depict their poor expression levels in both cell lines, as apparent from the lack of detectable bands in the western blots. (**b**) Whole cell lysates of K562 and KCL22 cells treated with imatinib (1μM), nilotinib (50nM) and dasatinib (3nM) for 18 and 36h were immunoblotted with the indicated antibodies, corresponding to the anti-apoptotic BCL-2 family members. (**c**) Same as **b,** but immunoblotted for the pro-apoptotic activators and effectors of the BCL-2 family. PUMA is poorly expressed and hence encircled. However, since a positive effect for PUMA was observed in TKI-induced apoptosis, the blots were repeated and presented immediately below with more protein (75μg) loaded in the gel to detect the bands corresponding to PUMA. (**d**) Western blots confirming the knockdown efficiency of siRNAs targeting mRNAs coding the indicated proteins. PUMA gels were run with more protein (75μg), as indicated above. (**e**) Same as **b**, but immunoblotted with the indicated antibodies, corresponding to the pro-apoptotic sensitizers. Bands corresponding to NOXA, HRK, BMF and BIK were barely detectable, and since HRK was the only protein that exhibited a positive effect in TKI-induced apoptosis, more protein (75μg) was loaded as indicated above, and presented immediately below the original blot. The asterisk in the HRK blots denotes a non-specific band that corresponds to an unidentified protein with a molecular weight of 16kD. (**f**) Western blots confirming the knockdown efficiency of siRNAs targeting mRNAs coding the indicated proteins. HRK, BMF and BIK were barely detectable in the gels.

**Figure S5.** Inhibition of BCL-X_L_ promotes rapid apoptosis in primary CML cells. (**a** and **b**) Diagnostic chronic phase CD34^+^ cells from low CIP2A patients were exposed to A-1331852 for 1h (n=3) and 4h (n=3) and apoptosis assessed by PS externalization.
